# Evaluating the Feasibility, Acceptability, and Preliminary Efficacy of SupportMoms-Uganda, an mHealth-Based Patient-Centered Social Support Intervention to Improve the Use of Maternity Services Among Pregnant Women in Rural Southwestern Uganda: Randomized Controlled Trial

**DOI:** 10.2196/36619

**Published:** 2023-03-02

**Authors:** Esther C Atukunda, Mark J Siedner, Celestino Obua, Angella Musiimenta, Norma C Ware, Samuel Mugisha, Josephine N Najjuma, Godfrey R Mugyenyi, Lynn T Matthews

**Affiliations:** 1 Mbarara University of Science and Technology Mbarara Uganda; 2 Department of Medicine and Center for Global Health, Massachusetts General Hospital Harvard Medical School Boston, MA United States; 3 Global Health and Social Medicine, Harvard Medical School Boston, MA United States; 4 Innovation Streams Limited (iStreams) Uganda Mbarara Uganda; 5 Division of Infectious Diseases School of Medicine University of Alabama at Birmingham Birmingham, AL United States

**Keywords:** mobile health app, mHealth app, feasibility and acceptability, messaging, health education, health promotion, app development, mobile phone

## Abstract

**Background:**

SMS text messaging and other mobile health (mHealth) interventions may improve knowledge transfer, strengthen access to social support (SS), and promote positive health behaviors among women in the perinatal period. However, few mHealth apps have been taken to scale in sub-Saharan Africa.

**Objective:**

We evaluated the feasibility, acceptability, and preliminary efficacy of a novel, mHealth-based, and patient-centered messaging app designed using behavioral science frameworks to promote maternity service use among pregnant women in Uganda.

**Methods:**

We performed a pilot randomized controlled trial between August 2020 and May 2021 at a referral hospital in Southwestern Uganda. We included 120 adult pregnant women enrolled in a 1:1:1 ratio to receive routine antenatal care (ANC; control), scheduled SMS text or audio messages from a novel messaging prototype (scheduled messaging [SM]), and SM plus SMS text messaging reminders to 2 participant-identified social supporters (SS). Participants completed face-to-face surveys at enrollment and in the postpartum period. The primary outcomes were feasibility and acceptability of the messaging prototype. Other outcomes included ANC attendance, skilled delivery, and SS. We conducted qualitative exit interviews with 15 women from each intervention arm to explore the intervention mechanisms. Quantitative and qualitative data were analyzed using STATA and NVivo, respectively.

**Results:**

More than 85% and 75% of participants received ≥85% of the intended SMS text messages or voice calls, respectively. More than 85% of the intended messages were received within 1 hour of the expected time; 18% (7/40) of women experienced network issues for both intervention groups. Over 90% (36/40) of the intervention participants found this app useful, easy to use, engaging, and compatible and strongly recommended it to others; 70% (28/40), 78% (31/40), and 98% (39/40; *P*=.04) of women in the control, SM, and SS arms, respectively, had a skilled delivery. Half (20/40), 83% (33/40), and all (40/40; *P*=.001) of the women in the control, SM, and SS arms attended ≥4 ANC visits, respectively. Women in the SS arm reported the highest support (median 3.4, IQR 2.8-3.6; *P*=.02); <20% (8/40; *P*=.002) missed any scheduled ANC visit owing to lack of transportation. Qualitative data showed that women liked the app; they were able to comprehend ANC and skilled delivery benefits and easily share and discuss tailored information with their significant others, who in turn committed to providing them the needed support to prepare and seek help.

**Conclusions:**

We demonstrated that developing a novel patient-centered and tailored messaging app that leverages SS networks and relationships is a feasible, acceptable, and useful approach to communicate important targeted health-related information and support pregnant women in rural Southwestern Uganda to use available maternity care services. Further evaluation of maternal-fetal outcomes and integration of this intervention into routine care is needed.

**Trial Registration:**

ClinicalTrials.gov NCT04313348; https://clinicaltrials.gov/ct2/show/NCT04313348

## Introduction

Antenatal care (ANC) is a mainstay for preventing maternal and perinatal morbidity and mortality, promoting the detection and treatment of prenatal complications, and identifying women at high risk to ensure delivery in skilled settings [1,2], but the use of these services in Uganda remains low. For example, only 58% of expectant mothers attend at least 4 ANC visits (of the recommended 8 by the World Health Organization) and only 70% of women deliver with a skilled attendant [3]. Consequently, Uganda has the highest maternal mortality (360 per 100,000 women) and child perinatal mortality rates (41 deaths per 1000 births) worldwide [3].

SMS text messaging and other mobile health (mHealth) interventions have been proposed to promote positive health behaviors and strengthen informed decision-making in women in the perinatal period [4-6]. Such interventions are hypothesized to improve outcomes through knowledge transfer and strengthened access to social support (SS). For example, mHealth interventions in pregnant women have been shown to increase ANC attendance [7,8], institutional delivery [9,10] and vaccination rates [4,10]. mHealth interventions that bolster SS can also improve pregnancy experiences by decreasing anxiety and depression [11-14], while increasing perinatal bonding [13] and communication [14]. These benefits are believed to be mediated through the promotion of family structure, partner involvement, and social networks, which in turn foster financial and emotional coping mechanisms to enable women to overcome socioeconomic and physical barriers to target outcomes such as food insecurity and transportation [14-17].

However, few mHealth apps for maternal care have been taken to scale in sub-Saharan Africa (SSA), where the contextual factors that drive successful interventions differ [18] but the public health impact of such interventions is likely to be the greatest. Some studies have hypothesized that the underutilization of behavioral science theory in intervention design contributes to the lack of successful interventions at scale [4,6]. Few apps incorporate end-user designs or iterative development. The Healthcare Utilization Model (HUM) highlights three dynamics that predict health care service use including (1) predisposing factors (eg, marital status, birth order, knowledge gap, and health beliefs), (2) enabling factors (eg, SS, community participation, information access, respectful patient-centered care, income, travel or waiting time, accessibility of ANC, and delivery services), and (3) perceived or evaluated needs (eg, the state and perceptions of current health or pregnancy and perceived benefits or threats) [19-25]. Although used to explain health-seeking behaviors in resource-rich countries [26,27], few studies have examined the HUM framework in low- and middle-income countries. Furthermore, there are no theory-informed mHealth interventions targeted at improving the use of maternity services by promoting SS.

We previously reported our iterative app development activities, including stakeholder interviews, content development, app design, and testing [28]. We now report the results of a pilot study to evaluate the feasibility, acceptability, and preliminary efficacy of this novel mHealth-based, patient-centered, and audio-based SMS text messaging app (*SupportMoms-Uganda*) that draws upon HUM concepts, mHealth technologies, and SS to communicate targeted health-related information and promote the use of maternity services by pregnant women in rural Southwestern Uganda.

## Methods

### Study Design

We conducted a 3-arm interventional study among pregnant women in Uganda to evaluate *SupportMoms-Uganda*, an mHealth app incorporating appropriate end-user intervention design characteristics, including SS network engagement through SMS text messaging notifications; motivators such as tailored, automated SMS text messaging; or voice call health information messaging to facilitate the uptake and use of maternity care services [28]. Scheduled SMS text messaging reminders were also incorporated as part of the intervention as a stimulus, prompt, or cue to take action. We used the behavioral change technique taxonomy [29,30] to identify and characterize the key components of this app aimed at communicating information on the benefits of nutrition, exercise, attending ANC, skilled delivery, partner involvement, birth preparedness, and monitoring danger signs. The app was designed using an end-user iterative approach to refine user-driven message content tailored to women’s needs and preferences. This trial was registered at ClinicalTrials.gov (NCT04313348).

### Study Participants

Two types of participants were enrolled in this study: (1) study participants, comprising pregnant women with a gestational age of £20 weeks (determined by the last menstrual period); and (2) their nominated social supporters. Eligible participants included (1) adults aged ≥18 years living in Mbarara district (within 20 km of the antenatal clinic), (2) having access to a mobile phone for personal use with reliable cellular phone reception, (3) being able to provide informed consent, and (4) willing to identify at least 2 social supporters or identified as a social supporter. We excluded women with known high-risk pregnancies at the time of enrollment, including hypertension; history of gestational diabetes and preeclampsia; or other severe birth complications because they could already be motivated to engage in ANC, and it would be unethical to enroll in the control group.

### Study Setting

This study was conducted at Mbarara Regional Referral Hospital, located approximately 290 km southwest of Uganda’s capital, Kampala. The hospital receives over 30,000 women attending routine ANC annually, including uncomplicated and high-risk pregnancies, and conducts over 12,000 deliveries annually. Maternity services, including delivery, are largely provided free of charge through public hospitals and health centers.

### Recruitment and Enrollment of Study Participants

Participants were screened for eligibility by a study nurse in the antenatal clinic and referred to a research assistant for enrollment or referred from village health teams [31]. Consenting participants were asked to identify at least 2 individuals from their existing SS network with whom they have had good, stable, and long-term relationships and believed they would be available to support them during the pregnancy and study follow-up period. Social supporters of at least 18 years of age, living within the same parish as the participant, who owned a cell phone for personal use with reliable cellular phone reception, and who knew the study participant’s pregnancy status were also eligible to enroll. Potential social supporters were excluded from the study if they were unable to use SMS text messaging or unwilling to receive SMS text message notifications. Potential social supporters were contacted during the first 2 weeks preceding participant enrollment to ensure an ongoing relationship at the time of their enrollment. The social supporters were then invited to participate in the study, consented, and enrolled. They were informed that they would receive weekly SMS text message notifications regarding the study participant’s next scheduled obstetric review during pregnancy and the postpartum period. No specific instructions or recommendations guiding social supporters on how to respond to SMS text message reminders were provided because the intervention was designed to build on existing supportive relationships among study participants. All participants provided written informed consent, or for those who could not write, a thumbprint was made on the consent form, as approved by the ethics committees. The study was conducted in a private space, and the data were coded and anonymous in accordance with the Declaration of Helsinki.

### Randomization and Blinding

Before study initiation, a study biostatistician digitally generated a random list used to determine arm assignment for study participants in block sizes of 20. Study participants were randomized equally in a 1:1:1 ratio to the control, scheduled messaging (SM), or SS arms. Once eligibility was established and participants consented to the study, a number was allocated by taking the next in a series of similar prior labeled opaque envelopes provided by the study coordinator to conceal group allocation. Research assistants were blinded to the study hypothesis as well as group allocation and were only informed of the arm assignment at the time of participant enrollment. Data were collected electronically. The data analyst was blinded to the group allocated to different study participants.

### Intervention Arms

Participants were screened for eligibility, randomized, and enrolled between August 2020 and May 2021 to one of three arms: (1) the standard routine care arm, (2) scheduled SMS text messaging arm, and (3) SS engagement arm. The standard routine care arm included routine information given to pregnant women at the maternity centers during ANC visits by clinic staff and midwives as per the Uganda’s Ministry of Health guidelines [32]. The scheduled SMS text messaging arm included automated health education SMS text messages or audio messages; a weekly SMS text message reminder about upcoming ANC appointments; and expected date of delivery at their preferred time, language, and day of the week. The content of the SMS text message reminders was customized and determined by participants at enrollment. If the participant had no preference, we used the message, “This is your ANC visit reminder, encouraging you to attend on [expected ANC visit date].” The SS engagement arm was similar to the scheduled SMS text messaging arm, with the addition of sending SMS text message reminders to the 2 participant-identified social supporters. Social supporters were also able to personalize the SMS text message content at enrollment; the default message read as follows: “We appreciate you being consistently close to your friend XX who is pregnant, we are reminding you of her upcoming antenatal visits on the date indicated on her card.” No additional health information was provided to the social supporters. Messages were sent as preferred in the local language Runyankole or English.

### Study Procedures

Participants in the intervention arms received SMS text messaging reminders, plus message content and information developed as part of the *SupportMoms-Uganda* app. The participants obtained instructions on how to use the app to retrieve or receive information. The times and lengths of the individual sessions were recorded and transmitted to the server. The phone served as a gateway to display and visualize the intended message content in the form of voice or text. Once reception or visualization was complete, data were transmitted from the gateway device to a secure web-based session and logged out to enable submission of data to the server for review via password access of any device that can access the web. Delays during periods of inadequate cellular reception were stored for later transmission. All study participants were given solar chargers and were reminded to charge their phones as needed during enrollment. App reception was considered a proxy for accessing information to alter existing predisposing factors (such as negative health beliefs) that could enable and improve the perceived need to seek care.

### Data Collection

All data collection was performed in Runyankole. Quantitative questionnaire data were collected from both study participants and their social supporters at enrollment on the following topics: sociodemographic characteristics, health, comorbidities and outcomes [33], food insecurity [34], SS [35], reproductive health history, and perceptions of pregnancy and delivery [36]. Reports of SS received by study participants did not specify the source as it could occur from outside the dyad studied here. Women were followed up for at least 6 months and, at exit (within 2-4 weeks following delivery), a survey was administered to assess the ability of participants to receive and understand SMS text messages or voice calls, technology usefulness, engagement, and acceptance. Exit questionnaires on feasibility and acceptability were developed using the Unified Theory of Acceptance and Use of Technology model [37]. Quantitative data were collected using a web-based database developed on *SupportMoms-Uganda* and ComCare platforms to improve data completeness, management, and quality control monitoring. A transport refund of US $3 was provided for each visit.

In addition, 30 face-to-face, in-depth exit interviews (15 from each intervention arm) were conducted to explore the patterns of SS and mechanisms of the intervention effect. Participants were purposively selected to ensure a range of prenatal and perinatal outcomes, types of SS reported, and intervention influence. These interviews were carried out within 2 to 4 weeks after delivery in a private place, lasted an hour, and were audio recorded. Interview guides were developed based on the observed quantitative results on technology acceptance. To maximize data quality, we asked interviewees to describe actual experiences and events whenever possible. Interview topics included (1) experiences with voice and SMS text messages received, including the most or least useful reminders, and communication between social supporters and study participants, including the things they talked about or did together; (2) acceptance and challenges, including mobile phone ease of use; their ability to understand, request, or receive support or guidance as needed; messaging problems experienced with the intervention; usefulness; and intention to use in future; (3) consequences, including changes or lack of changes resulting from the use of messages; and (4) comparisons and attitude, including differences, similarities, and attitude across the messaging types and suggested changes.

### Study Outcomes

Our primary outcome measures were (1) the feasibility of *SupportMoms-Uganda* app prototype, assessed by the number of received calls or SMS text messages and ability of participants to read or listen to and understand messages; (2) the acceptability of *SupportMoms-Uganda* app, measured using the Technology Acceptance Model to assess ease of use, motivation, social influence, perceived control, attitude toward use of the technology, and its usefulness; (3) average number of ANC visit attendance; and (4) proportion of births attended by a skilled provider. Other secondary outcomes include mode of delivery, maternal complications or need for resuscitation, birth weight, stillbirths, intrauterine fetal deaths, maternal deaths, interaction with a social supporter, and overall reported SS received by the study participant during pregnancy to improve her pregnancy experience and ANC visit attendance. SS was defined as (1) enabling the study participant to reach the clinic or hospital through monetary support, direct transportation, or taking care of daily activities while they are absent or (2) motivating the study participant to go for scheduled and necessary prenatal checkups, reviews, and skilled births, including addressing cognitive and behavioral barriers, such as food insecurity, depression, and alcohol use.

### Sample Size Estimation and Data Analysis

We determined our sample size for a 3-armed pilot randomized controlled trial intended to identify unforeseen app uptake and use problems among eligible pregnant women in Uganda. Using the rule of thumb [38,39], we calculated the sample size needed to identify 2.5% of social and technical problems that may arise among *SupportMoms-Uganda* users, with a 95% CI of 120 participants.

#### Quantitative Data Analysis

We used summary statistics to compare the health-related and sociodemographic data of study participants between arms; data specific to social supporters will be presented elsewhere. We also assessed the technical function of the intervention using the following statistics: number of successful calls, SMS text messages delivered and received by the participant over the number of SMS text messages or calls anticipated per protocol, number and type of technical problems encountered, number of SMS text message notifications sent to social supporters, actual messaging reception and use, number of women using the SMS text message response and interactive message feature, messages coming within 1 hour of expected time, reminder or notification, and total ANC reviews. We assessed acceptability by describing technology expectancy, skills, facilitating conditions, acceptance, and engagement as per the Unified Theory of Acceptance and Use of Technology model. Although not powered to detect significant differences, we compared technology and maternal health outcomes among the 3 study arms to explore group differences using 1-way ANOVA. Study participant’s SS was divided into instrumental (physical and economic) and emotional (emotional and informational) support. The Household Food Insecurity Access Scale was calculated as recommended [40], and the median score was considered the cut-off for food insecurity. Instrumental SS and food insecurity were described because of the low-resource nature of this setting, which may impede the ability to provide physical support despite the intention to do so. Data analysis was conducted using STATA (version 13; StataCorp).

#### Qualitative Data Analysis

Transcripts were generated from audio-recorded interviews. Qualitative data were coded using NVivo (version 12.0; QSR International) data management software. Coded data were iteratively reviewed and sorted to identify repeated themes (topics) arising from the data. Themes were generated using inductive content analysis [41]. Data analysis was performed jointly by ECA and JNN. Both JNN and ECA double-coded 5 sampled transcripts, yielding a Cohen κ statistic of 0.852. Together with EA, we resolved the coding disagreements to ensure consistency in the codebook. The content consisted of descriptive labels that defined and specified each theme’s meaning, along with illustrative quotes taken from the qualitative interviews.

### Ethics Approval

We formed an independent committee involving a biostatistician and clinicians with expertise in health service use and obstetric care. This committee, together with the community Advisory Board at Mbarara University, monitored participant confidentiality, data quality, implementation, outcomes, and potential harms. This study was reviewed and approved by the Mbarara University of Science and Technology Institutional Ethics Review Committee (registration number 13/09-18) and the Uganda National Council for Science and Technology, Kampala, Uganda (registration number SS 4809). Permission to conduct the study was obtained from district and local community leaders.

## Results

### Participant Characteristics

Of the 161 women screened for eligibility from July 12 to September 20, 2020, a total of 74.5% (n=120) of women were eligible, and all participants consented to participate in the study. A total of 120 women were randomized equally into 3 study arms: control, scheduled SMS text messaging, and SS. All participants completed the study procedures. Their demographic and clinical characteristics were similar across the 3 study groups (Table 1).

**Table 1:** Baseline demographic and clinical characteristics of women enrolled on the messaging app (N=120).

Characteristics		Control (n=40)	Scheduled messages (n=40)	Social support (n=40)
Age (years), mean (SD)		27.5 (4.3)	26.9 (3.8)	28.1 (3.5)
**Marital status, n (%)**				
	Married	25 (63)	27 (68)	25 (63)
	Single or separated	15 (38)	13 (33)	15 (38)
Educational attainment (primary), n (%)		14 (35)	16 (40)	17 (43)
**Parity, n (%)**				
	0	14 (35)	12 (30)	16 (40)
	1	9 (23)	10 (25)	11 (28)
	2	6 (15)	5 (13)	7 (18)
	≥3	11 (28)	13 (33)	6 (15)
Current pregnancy is planned, n (%)		27 (68)	30 (75)	28 (70)
History of still birth, n (%)		4 (10)	4 (10)	3 (8)
Anyone in household aware of the pregnancy, n (%)		37 (93)	36 (90)	36 (90)
Household income (Ugandan Shilling >150,000 [approximately US $42]), n (%)		25 (63)	23 (58)	20 (50)
Social support score^a^, median (IQR)		2.4 (2.1-2.8)	2.8 (2.3-3.1)	2.5 (2.2-3.0)
Food insecure (HFIAS^b^ >8), n (%)		11 (28)	13 (33)	11 (28)
History of complications in pregnancies, n (%)		6 (15)	5 (13)	6 (15)
**Mobile telecom service provider^c^, n (%)**				
	MTN (Mobile Telephone Network-Uganda)	30 (75)	28 (70)	30 (75)
	Airtel-Uganda	31 (78)	33 (83)	30 (75)
**Preferred time to receiving messages, n (%)**				
	Sunrise to midday	9 (23)	10 (25)	13 (33)
	Midday to sunset	6 (15)	5 (13)	5 (13)
	Sunset to midnight	5 (13)	7 (18)	10 (25)
	Anytime	20 (50)	18 (45)	12 (30)
**Preferred days of the week for messaging, n (%)**				
	Weekdays	11 (28)	9 (23)	8 (20)
	Any day or no preference	29 (73)	31 (78)	32 (80)

^a^This score ranges from 1 to 4, with 4 indicating high levels of social support.

^b^HFIAS: Household Food Insecurity Access Scale.

^c^Multiple responses.

### Primary Finding 1: Feasibility

At least 1 cell phone was reported in a household for both the scheduled SMS text messaging and SS arms (median 2, IQR 1-3), and 20% (8/40) reported smartphones (Table 2). More than 70% of the women in both groups owned a personal cell phone, and all women in both groups were able to operate the phone for either SMS text messages or voice calls. More than 85% of automated informational SMS text messages, >70% of automated audio messages, and >80% of SMS text message reminders were successfully sent throughout the study period for both intervention arms. At least 85% and 75% of participants received a minimum of 85% of the intended SMS text messages or voice calls, respectively. All participants received at least 65% and 60% of the intended SMS text messages or voice calls, respectively, for either group. At least 85% of all participants used the interactive or response-messaging feature of the app. The messaging interactive feature was rated good by at least 90% of the participants in both intervention groups. More than 85% of all messages were received within 1 hour of the expected time, with less than 20% of participants in both intervention groups reporting network issues as a reason for missing or delayed calls or messages. Using a scale of 1 to 5, all participants were able to hear calls clearly in both intervention groups, with on-call engagement lasting an average of 1.5 minutes. Confidentiality was ranked as the least important feature of a cell phone on SMS text messaging; clarity and language of the message was ranked as important for both groups.

**Table 2:** Feasibility and use of the SupportMoms-Uganda messaging app.

Characteristics		Scheduled messaging arm (n=40)	Social support arm (n=40)
**Number of accessible mobile phones^a^**			
	Household, median (IQR)	2 (1-3)	2 (1-3)
	Neighborhood, median (IQR)	3 (2-4)	3 (2-5)
	Smartphones, n (%)	8 (20)	8 (20)
Owns a personal cell phone, n (%)		30 (75)	31 (78)
Ability to operate phone for SMS text messaging or voice call, n (%; 95% CI)		40 (100; 91.2-100)	40 (100; 91.2-100)
**Total number of messages sent automatically, n (%; 95% CI)**			
	Informational SMS text messaging	2683 (86.02; 84.80-87.22)	2702 (86.71; 85.53-87.90)
	Audio messages	752 (72.3; 69.5-75.0)	783 (75.3; 72.6-77.9)
	Participant SMS text messaging reminders	646 (80.8; 77.8-83.4)	1312 (82; 80.01-83.86)
	Supporter SMS text messaging reminders	N/A^b^	671 (83.9; 81.1-86.4)
**Number of planned messages received, n (%; 95% CI)**			
	≥85% SMS text messages	34 (85; 70.2-94.3)	36 (90; 76.3-97.2)
	≥85% voice calls	30 (75; 58.8-87.3)	33 (83; 67.2-92.7)
	≥65% SMS text messages	40 (100; 91.2-100)	40 (100; 91.2-100)
	≥60% voice calls	40 (100; 91.2-100)	40 (100; 91.2-100)
Messages came within 1 hour of expected time, n (%; 95% CI)		34 (85; 70.2-94.3)	35 (88; 73.2-95.8)
**Response messaging feature, n (%; 95% CI)**			
	Used the response feature	34 (85; 70.2-94.3)	35 (88; 73.2-95.8)
	Rate response feature >3	36 (90; 76.3-97.2)	37 (93; 79.6-98.4)
**Reasons for missed or delayed messages^a^, n (%; 95% CI)**			
	Sometimes turn off phone deliberately	6 (15; 5.7-29.8)	4 (10; 2.7-23.7)
	Network issues	7 (18; 7.3-32.8)	5 (13; 4.2-26.8)
	Battery or phone charging issues	3 (8; 1.6-20.4)	1 (3; 0.1-13.2)
	Lost phone or phone functionality	6 (15; 5.7-29.8)	3 (8; 1.6-20.4)
Rate ability to hear voice message clearly >3, n (%; 95% CI)		40 (100; 91.2-100)	40 (100; 91.2-100)
Average length of on-call engagement (minutes), mean (SD)		1.5 (0.3)	1.5 (0.2)
**Ranking important features of messaging^c^, n (%; 95% CI)**			
	Confidentiality: least important	40 (100; 91.2-100)	40 (100; 91.2-100)
	Clarity of message: most important	40 (100; 91.2-100)	40 (100; 91.2-100)
	Language: most important	36 (90; 76.3-97.2)	36 (90; 76.3-97.2)

^a^Multiple responses.

^b^N/A: not applicable.

^c^Scale of 1 to 5, with 1 as least and 5 as greatest.

### Primary Finding 2: Acceptability

As shown in Table 3, >90% of app users found it generally acceptable and helpful. All participants found the messaging app useful, motivating, and improved their involvement in health matters that concern them. Nearly all participants found the messaging app clear and easy to use and the content of the messaging program easy to understand. All participants liked the messaging program and found it fun and interesting; nearly all women obtained support from people who influenced their behavior or those around them to use the messaging program. More than 80% of women did not need additional resources to use the messaging program for both the SM (32/40, 80%) and SS (37/40, 93%) groups. All women had the knowledge necessary to use the messaging program, and none reported incompatibility of the program with their existing messaging programs on their phones. Nearly all women reported to have had enough skills to operate the phone for all SMS text message or voice calls. None reported anxiety or apprehension, fear, intimidation, or hesitation to use the messaging program. All women intended, predicted, and planned to use the messaging program in the future and would definitely recommend it to others. Using a scale of 1 to 5, at least 90% of the participants rated SMS text messaging and voice messages as highly relevant for both groups. None of the women found the scheduled SMS text messages or voice calls bothersome but engaging. Compared with routine calls or SMS text messages, >95% of participants in both groups read SMS text messages or received voice calls in the app whenever they saw them all the time. All interviewed participants found the messaging program convenient. Additional details are presented in Table 3.

**Table 3:** Acceptability of the SupportMoms-Uganda messaging app following the Unified Theory of Acceptance and Use of Technology model (N=40).

		Scheduled messaging (n=40), n (%; 97.5% CI)	Social support (n=40), n (%; 97.5% CI)
**Performance expectancy**			
	I find the messaging program useful in my pregnancy journey	40 (100; 91.2-100)	40 (100; 91.2-100)
	Using the messaging program motivates me to attend to my ANC^a^ visits seriously	40 (100; 91.2-100)	40 (100; 91.2-100)
	Using the messaging program increases my involvement in health matters that concern me	40 (100; 91.2-100)	40 (100; 91.2-100)
	Rate^b^ usefulness of the messaging program ≥4	40 (100; 91.2-100)	40 (100; 91.2-100)
**Effort expectancy**			
	My interaction with the program is clear and understandable	39 (98; 86.8-99.9)	40 (100; 91.2-100)
	Messaging program was easy to use	40 (100; 91.2-100)	40 (100; 91.2-100)
	Learning to operate the program was easy for me	40 (100; 91.2-100)	40 (100; 91.2-100)
	Rate^b^ ability to understand the message content >3	39 (98; 86.8-99.9)	38 (95; 83.1-99.4)
**Attitude toward the messaging program**			
	Working with the messaging program is fun	36 (90; 76.3-97.2)	40 (100; 91.2-100)
	I like working with the messaging program	40 (100; 91.2-100)	40 (100; 91.2-100)
**Social influence**			
	People who influence my behavior think that I should use the messaging program	40 (100; 91.2-100)	40 (100; 91.2-100)
	People around me have supported use of messaging program	36 (90; 76.3-97.2)	40 (100; 91.2-100)
**Facilitating conditions**			
	I have the resources necessary to use messaging program	32 (80; 64.4-91.0)	37 (93; 79.6-98.4)
	I have the knowledge necessary to use program	40 (100; 91.2-100)	40 (100; 91.2-100)
	Program not compatible with other messaging programs I use.	0 (0; 0-8.8)	0 (0; 0-8.8)
**Self-efficacy**			
	I could complete a job or task with no one to tell me what to do	39 (98; 86.8-99.9)	40 (100; 91.2-100)
**Anxiety**			
	I feel apprehensive about using the messaging	0 (0; 0-8.8)	0 (0; 0-8.8)
**Behavioral intention to use messaging program**			
	I intend to use the program in future	40 (100; 91.2-100)	40 (100; 91.2-100)
	I plan to use the program in future	40 (100; 91.2-100)	40 (100; 91.2-100)
	I definitely recommend it to others to use	40 (100; 91.2-100)	40 (100; 91.2-100)
	Rate^b^ relevance of the messages >3	39 (98; 86.8-99.9)	40 (100; 91.2-100
**Technology engagement or fatigue (compared with the traditional SMS text messages or voice calls)**			
	I found the study messages bothersome	0 (0; 0-8.8)	0 (0; 0-8.8)
	I found the voice messages engaging	36 (90; 76.3-97.2)	40 (100; 91.2-100)
	I found the SMS messages engaging	32 (80; 64.4-91.0)	40 (100; 91.2-100)
	I always receive these calls whenever I see them	38 (95; 83.1-99.4)	40 (100; 91.2-100)
	I read these SMS whenever I see them: all the time	40 (100; 91.2-100)	40 (100; 91.2-100)
Rate^b^ convenience of the messaging program >3		40 (100; 91.2-100)	40 (100; 91.2-100)

^a^ANC: antenatal care.

^b^Scale of 1 to 5, with 1 as least and 5 as greatest; proportions of 100% and 0% have been calculated using 1-sided 97.5% CIs.

### Preliminary Birth Outcome Data by Arm

In total, 88% (35/40), 90% (36/40), and 88% (35/40) of women delivered vaginally in the control, scheduled SMS text messaging, and SS arms, respectively (Table 3); 70% (28/40), 78% (31/40), and 98% (39/40; *P*=.04) of women in the control, SM, and SS arms, respectively, had a skilled delivery; 50% (20/40), 83% (33/40), and 100% (40/40; *P*=.001) of women in the control, SM, and SS arms attended ≥4 ANC visits, respectively. Although we noted fewer maternal complications or needs for neonatal resuscitations, stillbirths, or intrauterine fetal deaths at delivery reported by women in the intervention groups than in the control group, the differences were not significant (*P*=.18). The types and extent of SS as reported by the study participants varied. Women in the SS arm reported the highest SS (median 3.4, IQR 2.8-3.6) compared with 2.8 (IQR 2.6-3.2) and 2.4 (IQR 2.2-2.8; *P*=.02) in the SM and control arms, respectively. More women in the SS group also communicated and interacted at least once a week with their social supporters about ANC and pregnancy needs (31/40, 78%; *P*=.002) compared with 40% (16/40) and 35% (14/40) in the SM or control arms, respectively. More study participants in the control arm (16/40, 40%; *P*=.01) did not interact with their social supporters about ANC and pregnancy needs compared with the other 2 intervention groups. Fewer participants missed scheduled ANC appointments in the SS group (8/40, 20%; *P*=.002) because they could not afford transport compared with women in the SM arm (23/40, 58%) and control arm (27/40, 68%; Table 4).

**Table 4:** Exploratory maternal outcomes of SupportMoms-Uganda messaging app (N=40).

Outcomes		Control arm (n=40)	Scheduled messaging arm (n=40)	Social support arm (n=40)	*P* value
Spontaneous vaginal delivery, n (%)		35 (88)	36 (90)	35 (88)	.82
Skilled delivery, n (%)		28 (70)	31 (78)	39 (98)	.04
Attended ≥4 ANC^a^ visits, n (%)		20 (50)	33 (83)	40 (100)	.001
Maternal complications or need for resuscitation, n (%)		8 (20)	5 (13)	3 (8)	.18
Birth weight <2.5 kg, n (%)		9 (23)	6 (15)	6 (15)	.42
Stillbirths or intrauterine fetal death at delivery, n (%)		3 (7)	0 (0)	0 (0)	.18
Reported maternal deaths, n (%)		0 (0)	0 (0)	0 (0)	N/A^b^
Overall reported social support, median (range)		2.4 (2.2-2.8)	2.8 (2.6-3.2)	3.4 (2.8-3.6)	.02
**Participant interaction with a social supporter about ANC and pregnancy needs, n (%)**					
	At least once a week	14 (36)	16 (40)	31 (78)	.002
	More than a week to a month	10 (25)	17 (43)	9 (23)	.17
	Never	16 (40)	7 (18)	0 (0)	.01
**Missed a scheduled appointment, n (%)**					
	Could not afford transport	27 (68)	23 (58)	8 (20)	.04
	Could not miss work	4 (10)	3 (8)	0 (0)	.18
	Could not get someone to leave family or children	7 (18)	4 (10)	1 (3)	.08

^a^ANC: antenatal care.

^b^N/A: not applicable.

### Qualitative Results

#### Overview

Of the 30 women interviewed, 20 (67%) had had a skilled delivery, 25 (83%) had attended at least 4 ANC visits, and 22 (73%) reported moderate to high SS. All non–control group participants received at least 50% of the planned SMS text messages or voice messages. Technology expectancy, acceptance, and engagement was dynamic across both intervention groups. Women reported different motivations, goals, likes, needs, and expectations while using or engaging in the messaging program that was customized and automated to deliver tailored audio messages or SMS text messages. From the qualitative data, all women described the intervention as useful, actionable, and easy to use; the tailored health information helped them to learn, internalize, and comprehend ANC and skilled delivery benefits, strengthening their informed decision-making as they were reportedly able to easily share and discuss information with their significant others, who in turn committed to providing them the needed support to prepare and seek help. Women identified 5 important app attributes that enabled them to use the program continuously. Women reported that they were able to (1) receive and understand messages easily and independently; (2) receive trusted and actionable information sent directly on mobile phones, which helped women pay more attention; (3) appreciate scheduled, personalized, and precautionary messages delivered in a friendly tone; (4) obtain complementary educational support for sharing with their friends and partners or for future reference; and (5) engage partners and social networks for needed support.

#### Receiving and Understanding Messages Independently

Women found the app familiar and easy to use. They reported receiving voice calls or SMS text messages on their or significant others’ phone devices effortlessly without added cost or skill. Because phones are familiar and already integrated into daily routines, women often use this technology. The expectations of using familiar devices also eased anxiety, apprehension, hesitation, or intimidation about the technology used for this messaging program. Their familiarity with cell phones improved their interaction with the messaging app, improved their understanding, and improved their behavioral intention to use the messaging program continuously and in the future:

I have always received calls or text messages so I was not worried at all. It was easy for me to follow texts and calls since it was the same number and code that sent those messages so I could easily identify and receive the messages whenever they came in.

29-year-old mother of 2 children whose last delivery was from a facility

I had no problem at all because I always use my phone for texting or calls...they came in with a specific number once a week so I was always able to read them easily and understand them even if my husband wasn’t around.

19-year-old mother of 1 child

#### Trusted and Actionable Information Sent Directly on Mobile Phones Helped Women Pay More Attention

Women reported that interacting with the app messages prompted them to take action as they were able to obtain trusted and credible information sent directly on their mobile phones. Women relied on information from the app to process and gain knowledge, as well as make informed decisions that would help them work through certain set maternity goals, such as delivering a healthy baby, having a safe skilled delivery, and attending scheduled ANC visits. Women also indicated that they were able to obtain useful and actionable information on health, instructions about different preferred topics, or how to perform safe motherhood behavior, as well as information on health consequences or regrets of poor health-seeking behavior, what to do, or where to seek care or redress to prepare and solve their identified problem, which kept them attentive and motivated. Routine information from a trusted source also helped women build confidence in the app and stay alert in reviewing their progress toward individual birth goals as they continuously interacted with their partners and health care providers for redress or follow-up on ANC monitoring visits:

I trusted the doctor that was teaching us on phone...she spoke to me so gently and I felt that she was calling and messaging me because she wished me and my baby well, so I was encouraged to always listen in. I learnt important things personally directly on my phone from a real doctor...I was encouraged to follow these instructions, go to hospital and seek help just in case.

25-year-old mother of 1 child

Sometimes, I would listen to the midwife at the health center but I often forgot everything...You hear so many things from relatives and friends and you don’t know what to do or who to trust. I am excited to always receive these messages from a place I trust directly on my phone and this always helps me to plan and review how I feel or how I am doing. I like them a lot. They keep me alert.

36-year-old mother of 5 children

#### Scheduled, Personalized, and Precautionary Messages Delivered in a Friendly Tone

App messages were preferred and expected at certain times of the day and week. Women reported that this interface helped them not to miss calls or texts unnecessarily or waste time waiting around expecting the messages. Unlike the random, redundant, and *unsolicited* messages routinely sent to their phones in different numbers by telecom companies, these scheduled messages from a known sender helped women plan and engage with the expected messages. These schedules helped women stay expectant, light, and excited to receive messages addressed to them. Customization with individual names, plus tailoring of these messages to their needs and demands, made the messages relevant, making women feel a sense of comfort and value. Women also felt included and understood by the app callers and promoters:

I received messages with my name on it and it made me feel important. It was exciting for me and I always felt good talking to my doctor on phone every Monday morning when she called. It was as if we were talking directly to each other and understood each other...Even my husband knows the number and time when messages come in so he always calls to remind me.

27-year-old mother of 2 children

I was happy someone included me in this plan to receive these important messages in my local language every Friday evening when I am not busy...It feels good and at least I don’t have to waste my time listening to those annoying and useless telecom messages that are randomly sent to my phones whenever they want.

33-year-old mother of 3 children

Women also described the app messages as precautionary and friendly and that such straightforward messages encouraged, motivated, and prompted them to take or plan actions, such as attending scheduled visits, seeking financing to prepare, or seeking skilled delivery. Information cues such as danger signs during pregnancy, communicated in a friendly tone, were said to help women appreciate their risks and keep them interested. Women also seemed to build more trusting relationships with their health care providers, as they engaged with the messaging app and continuously understood birthing procedures or processes through these cautionary messages. This continuous engagement helped women seek formal maternity care services that facilitated more useful one-on-one information transfers and support. Notably, the delivery of these messages was reported to be friendlier and more responsive to their needs compared with routine group health education experiences at public health facilities:

You know many times we go through a tough day and even if one is helping you understand difficult things, one needs that one-on-one voice that sounds gracious and encouraging...The doctor on phone always talked to me in a friendly way, as if we have met before. It goes along way and she made me interested in whatever she was telling me. I do not know her but she sounded serious but caring.

21-year-old mother of 1 child

The messages were always clear and to the point especially on what could happen to you and what to do to avoid danger...I always waited for the last part (laughter) that sounded like a caution or a strong warning for danger, but in a good way and many times it prompted me to check myself every time, or go to see my doctor to help me understand my situation if I was in doubt.

37-year-old mother of 2 children

#### Continuous and Complementary Educational Support for Sharing or Future Reference

Women described the app as a good and ongoing way of obtaining information that they stored on their phone for future reference or sharing with friends within their social networks. Some women described this continuous and customized messaging approach as continuity of care and the needed confidence in the intervention as a “birth companion” that helped them learn, keep motivated, and monitor their progress in time. Sharing this information with others was also reported to improve interaction and engagement with the app and others, such as women, and reviewed and shared knowledge. The ability to receive, understand, store, and share information with peers, spouses, and significant others from a *credible* source was seen as a more important factor than the actual provision of messages, which reportedly empowered women through call back, repeat, or other app interaction features:

I still have my messages on my phone and I can read them or share them with other friends in my group...People know I have credible information and that doctors in your program contact me directly on my phone so they can ask me when we talk. It feels good to always have this information on your journey and you are not confused by anyone anyhow.

30-year-old mother of 1 child

We don’t get any written information from anywhere, including health centers. I got an opportunity to get good information that I can keep on my phone and read later whenever I want to...Imagine I also got reminded when to prepare to go to hospital every time...it’s like the best companion for such a long birthing journey. It’s a good thing. I loved it.

25-year-old mother of 2 children

#### Engagement of Partners and Social Networks for Needed Support

Active engagement of women and their social supporters through SMS text messaging reminders or their phones to access important, targeted health information during pregnancy helped women mobilize the needed company and resources to access maternity services: fairly or adequately preparing for birth. Women reported that partner engagement in the messaging program improved their involvement and communication as they sought to understand their risk or schedule and offered the necessary physical, financial, and emotional help to seek care in time before complications occurred. The approach to independently consent to these social supporters in the study seemed crucial to reinforce technology trust and confidence. The active involvement of preferred social supporters in the messaging program was reported to improve their physical interaction about pregnancy needs as well as the quality of women’s pregnancy and birthing choices and experiences:

Whenever I received those messages, my husband or sister contacted me immediately...sometimes they ask me when next I will be going to hospital and what I need so we get to talk and interact about that stuff often...we have bonded in our journey and he is always curious to listen to these messages, or read the texts and ask how I am doing or what I need every time it comes in.

29-year-old mother of 3 children

My husband got to learn these things with me and he helps me out a lot these days...He always complained not to have time to go with me. It was important to send him these messages because he now looks to understand more on why I need to go to hospital often and we don’t argue a lot when I need to go.

36-year-old mother of 4 children

## Discussion

### Principal Findings

We assessed the feasibility, acceptability, and preliminary effectiveness of a novel, patient-centered, and audio-based SMS text messaging app to support women in using maternity care services in rural Southwest Uganda. We observed high intervention acceptability and feasibility, with >80% of women receiving ≥85% of the intended messages within 1 hour. More than 90% of the women found this intervention useful, easy to use, interesting, appropriate, engaging, and compatible and would strongly recommend it to others. Nearly all women (39/40, 98%) in the SS arm had a skilled delivery compared with 78% (31/40) and 70% (28/40) in the SM and control groups, respectively. All women whose social supporters were engaged in the app attended ≥4 ANC visits, compared with 83% (33/40) and 50% (20/40) of women in SM and routine ANC, respectively. More study participants in the control arm (16/40, 40%; *P*=.01) did not interact with their social supporters about ANC and pregnancy needs compared with the other 2 intervention groups. Fewer women in the SS arm (8/40, 20%; *P*=.002) missed any visits owing to lack of transportation, compared with 58% (23/40) and 68% (27/40) of women in the SM and routine care arms, respectively. Women in the SS arm reported improved SS (3.4, IQR 2.8-3.6) compared with 2.8 (IQR 2.6-3.2) and 2.4 (IQR 2.2-2.8; *P*=.02) in the SM and control arms, respectively. The interactive messaging feature was rated highly by >90% of participants in both intervention groups. Our screen-to-eligible (120/161, 74.5%) and eligible-to-enroll (120/120, 100%) ratios were very high, suggesting promise or potential for wide reach. None of the participants were lost to follow-up. Our pilot data support the first mHealth app developed in the SSA setting by the SSA team to leverage existing social networks to support SSA women with promising findings.

In the qualitative interviews, all women described the intervention as useful, actionable, and easy to use; it helped them learn, cope, prepare, and take action within a friendly, trusted, and familiar environment. Scheduled, customized, and precautionary messages delivered in a friendly tone at preferred times of the week were valued as motivating and encouraging. The app was reported to provide complementary educational support for future reference or for sharing among their social networks. Women expressed that tailored health information helped them to learn, internalize, and comprehend ANC and skilled delivery benefits. This strengthened their informed decision-making, as they were reportedly able to easily share and discuss information with their significant others, who in turn committed to providing them the needed support to prepare and seek help. Women also expressed that involvement of their significant others within a friendly, trusted, and familiar environment helped them to mobilize needed support during pregnancy. Involving both health care providers and end users in characterizing, developing, and formulating the mHealth intervention allowed tailoring the intervention to their preferences. We incorporated women’s expectations, experiences, perceptions, and choices of a familiar mHealth-based technology that would benefit and support them in seeking professional maternity care within their local communities long-term, subject to the standard limitations of mobile phone ownership, type, and network challenges in the region.

Prior studies have reported improved engagement, acceptability, and use of programs that have been developed using a patient-centered approach, where mHealth interventions aim to address barriers to health care use through a multipronged approach by (1) teaching positive health behaviors and addressing specific health concerns (predisposing factors), (2) empowering and strengthening informed decision-making (enabling factors), and (3) improving the perceived need for the use of available services [4-6,18,42-46]. Such novel mHealth interventions help individuals internalize the benefits of health services and strengthen informed decision-making, especially when provided with adequate and relevant information on the promoted behavior to reduce the risk of morbidity and mortality, support healthier lifestyles, empower, and enable individuals to address specific health concerns or seek help [47]. This patient-centered mHealth intervention offered women complementary support through mobile phones as a health communication tool to bridge information gaps and provide continuity of care through tailored and targeted messaging.

Many women in Uganda are largely dependent on their significant others for economic provision, which together with the existing gender and traditional norms and beliefs, limits women’s ability and freedom to make family or health decisions to seek skilled care [31,48]. Knowledge gaps majorly influenced women’s past and future decisions to not attend ANC and pursue unskilled home births [31,48]. In line with previous studies [4-6,17,42,47,49,50], our ongoing and directed engagement and support at individual and family or societal levels were observed to have meaningfully or significantly improved individual risk internalization, partner involvement, pregnancy experience, perceived need, and informed decision-making to attend scheduled ANC visits and deliver in the presence of a health care provider. Similar directed and customized mobile phone–based interventions have previously been observed to motivate and inspire women, as well as offer individual or family SS [11-14,51], cues to action [52], or a source to challenge and debunk societal negative beliefs influencing access and use of health care [53].

With increasing and changing demands, tastes, trends, and preferences, users need relevant, appealing, and unique approaches and not the *one-size-fits-all* approach. The *SupportMoms-Uganda* app used theoretical models to develop appropriate and high-yield intervention design characteristics, such as an easy-to-use interface; use of familiar technology; SS network engagement through automated SMS text message notifications; motivators such as tailored SMS text messaging, voice information messaging, or customized reminders; and key factors that jointly improve participant experience and facilitate the use and retention of the messaging program. Women reported that this program was relevant and useful when personalized to fit their needs and demands. Our data also showed that our multifaceted mobile app designed using a behavioral model improved the use of maternity services, especially among women in the SS group who continuously shared their experiences concerning their milestones, concerns, challenges, and goal attainments with their significant others. Such ongoing sharing and feedback experiences that involve health care providers and significant others toward the attainment of set goals and targets have been documented to motivate app users [54]. In line with previous studies, characterizing the key components of an intervention, tailoring, and personalizing the information for end users improves engagement, ownership, motivation, and use of the intervention [29,30,54]. Previous studies have found that the SMS text message language, medium of message delivery, experience with similar technology, phone type, and characteristics are critical in designing and delivering a culturally appropriate mHealth program [18,44]. The engagement of social networks through SMS text message reminders has been documented to motivate individuals toward positive health behaviors and provide active SS to access health services [4-6,17,42]. Scheduled SMS text message reminders (1-way SMS text message sent on a fixed schedule, such as daily and weekly) and telephone apps have also been said to work as support, incentive, or enablers, especially when provided with accurate and relevant information on the promoted behavior [47]. Scheduled and automated messages help avoid technology fatigue, unnecessary repetition, and burdensomeness, making the messaging intervention an acceptable tool for delivering health promotion content [55]. SMS text messaging and voice calls for pregnant women have also been documented to increase singular maternal and child health outcomes, such as ANC attendance [7,8], institutional delivery [9,10], and vaccination rates [4,10] in low- and middle-income countries.

Our study had several strengths. Our app integrated maternal health epidemiology, well-established behavioral change theories, and qualitative research methods to characterize and consider key components of a patient-centered messaging app to improve maternity care use, making our findings more grounded, meaningful, acceptable, and generalizable in similar settings. We used both qualitative and quantitative methods to investigate the synergistic impact of a combination of novel mHealth interventions comprising SMS text message reminders and health information and leveraging SS to empower and motivate women to access perinatal care and improve maternal child health in the region. We used a stepped multidisciplinary approach that improved technology ownership, inclusiveness, confidence, and uptake to improve maternity service use among the targeted end users. Our research findings provide preliminary data that can be used to perform power calculations for a phase 3 definitive randomized controlled trial to evaluate the effectiveness of the *SupportMoms-Uganda* app or intervention compared with routine care in improving maternal and child health outcomes in Uganda.

Our study has some limitations. Many people in Uganda are transient in searching for stable work or new settlements [17], including during pregnancy. Some participants changed or lost their mobile phones or had inaccessible phones because of network issues. In addition, travel was restricted during the COVID-19 pandemic lockdowns, which might have affected the travel and attendance of ANC. However, these effects were distributed across all 3 arms. We leveraged our previous clinical research experience to maximize retention in care through the enrollment and engagement of alternative contacts in their social networks. We also actively explained the study purpose, schedule, and expectations at the time of enrollment and updated the residence and phone details at each follow-up visit to minimize lost to follow-up. We used appropriate means of contact based on participant preferences and information on the best telephone network for the time of the day to call or send text messages. Although we emphasized that participation in this study was voluntary, no eligible participant declined or withdrew from the study. Our study setting includes mainly persons from a less affluent or educated background and fewer smartphone users, limiting internet access despite improved internet penetration through local mobile phone companies. As such, the messaging content and delivery medium was developed to suit most phone types and characteristics for similar settings, and thus findings or the approach might not be generalizable to settings where literacy, the internet, or smartphone use is high.

### Conclusions

Our study contributes to a greater understanding of the characteristics and complexity of mHealth messaging apps that leverage SS networks and relationships to influence the uptake and use of maternity services. We demonstrate that developing a novel, patient-centered, and customized audio-based SMS messaging app is a feasible approach to communicate important targeted health-related information and support rural pregnant women in Southwestern Uganda to attend scheduled ANC visits and deliver them in the presence of a skilled health care provider. We also demonstrated that developing a useful and appropriate patient-centered, audio-based SMS text messaging app is widely acceptable in Southwestern Uganda to support women in accessing timely and credible health-related information through targeted and customized mHealth messaging approaches sent directly to individual mobile phones. We observed that involving end users gave women an opportunity to develop a tailored app according to their needs, preferences, and demands, an approach that was seen to improve technology engagement, as well as the uptake and use of available maternity care services. Women liked the app and described it as useful and easy to use, helping them learn, cope, prepare, and take action in a friendly atmosphere. Further evaluation of the effectiveness and integration of this mHealth-based SS intervention into routine care as a novel approach to improving maternal-perinatal health outcomes in Uganda is needed.

## Appendix

Multimedia Appendix 1 CONSORT eHEALTH checklist (V.1.6.1).

## Abbreviations

ANC: antenatal care
HUM: Healthcare Utilization Model
mHealth: mobile health
SM: scheduled messaging
SS: social support
SSA: sub-Saharan Africa
